# Supplementary material for: From sequence to enzyme mechanism using multi-label machine learning
Source: BMC Bioinformatics. 2014 May 19;15:150. doi: 10.1186/1471-2105-15-150 (PMC4229970; doi:10.1186/1471-2105-15-150)
Supplement: Additional file 2 — Java code of ml2db. Additional file ml2db_code.tar.gz contains the Java source code to run the multi-label machine learning experiments and save the results to database. The code’s Javadoc is included. [file 1471-2105-15-150-S2.zip › additional file 2/ml2db/ml2db/doc/mulan/classifier/lazy/class-use/MultiLabelKNN.html]

Uses of Class mulan.classifier.lazy.MultiLabelKNN


JavaScript is disabled on your browser.


- Overview
- Package
- Class
- Use
- Tree
- Deprecated
- Index
- Help

- Prev
- Next

- Frames
- No Frames

- All Classes

## Uses of Class mulan.classifier.lazy.MultiLabelKNN

- Packages that use MultiLabelKNN

  | Package | Description |
  | --- | --- |
  |  |  |
  | --- | --- |
  | mulan.classifier.lazy |  |
- - ### Uses of MultiLabelKNN in mulan.classifier.lazy

    Subclasses of MultiLabelKNN in mulan.classifier.lazy

    | Modifier and Type | Class and Description |
    | --- | --- |
    |  |  |
    | --- | --- |
    | `class` | `BRkNN` Simple BR implementation of the KNN algorithm.For more information, see |
    | `class` | `IBLR_ML` This class is an implementation of the "IBLR-ML" and "IBLR-ML+" methods for the MULAN package.    For more information, see    Weiwei Cheng, Eyke Hullermeier (2009). |
    | `class` | `MLkNN` Class implementing the ML-kNN (Multi-Label k Nearest Neighbours) algorithm.    For more information, see    Min-Ling Zhang, Zhi-Hua Zhou (2007). |

- Overview
- Package
- Class
- Use
- Tree
- Deprecated
- Index
- Help

- Prev
- Next

- Frames
- No Frames

- All Classes
